# Radiofrequency Ablation of Papillary Thyroid Microcarcinomas

**DOI:** 10.1016/j.aace.2022.02.005

**Published:** 2022-02-16

**Authors:** Leonardo Guimarães Rangel, Jose Higino Steck, Erivelto Martinho Volpi, Jonathon O. Russell, Ralph P. Tufano

**Affiliations:** 1 State University of Rio de Janeiro, Rio de Janeiro, Brazil; 2 Campinas State University, São Paulo, Brazil; 3 Oswaldo Cruz German Hospital, São Paulo, Brazil; 4 Johns Hopkins University, Baltimore, Maryland

**Keywords:** RFA, radiofrequency ablation

### Case Presentation

**Fig. 1:**
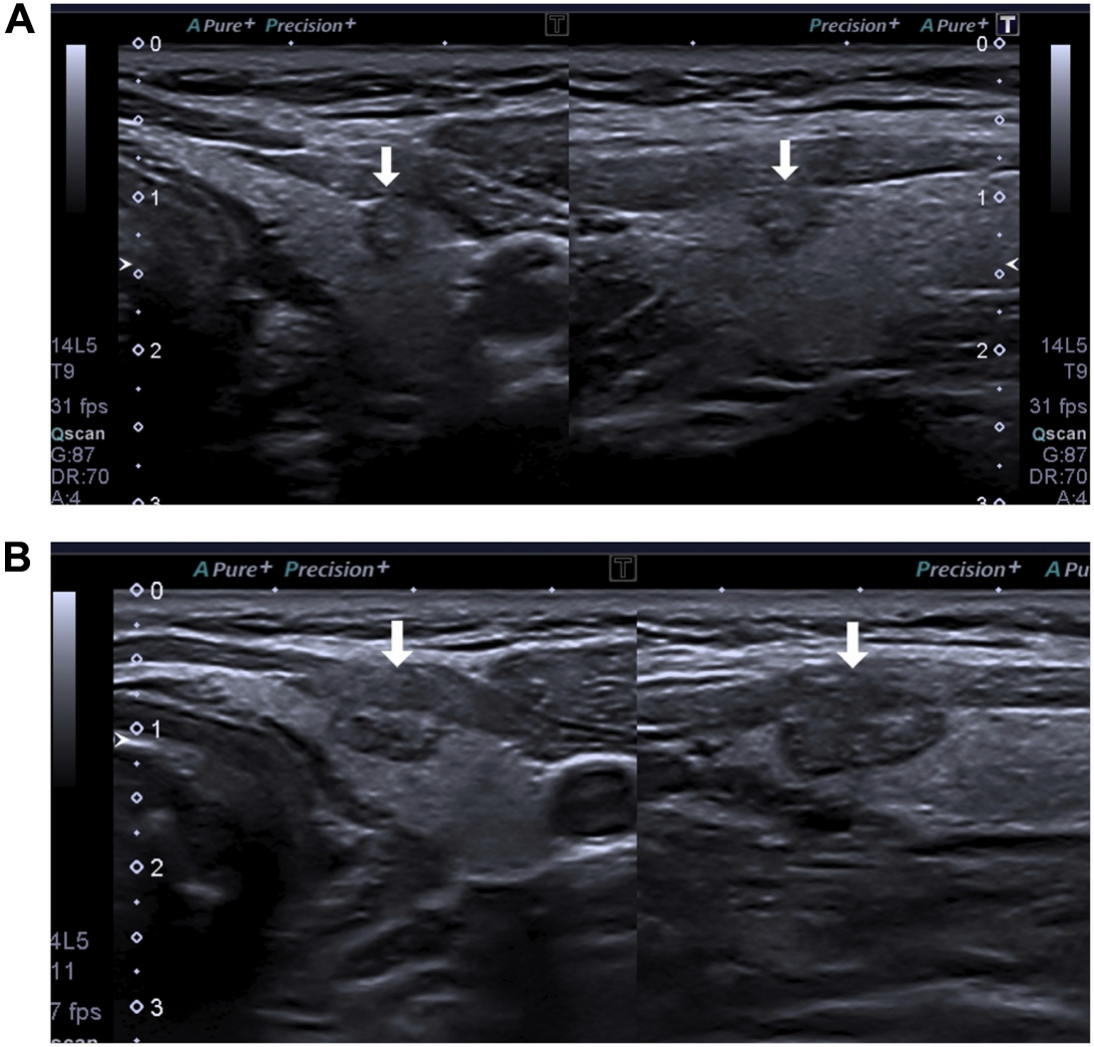

A 33-year-old obese woman presented to the endocrinologist looking for weight loss treatment and consultation. During the investigation, albeit the normal thyroid function, thyroid ultrasound was performed as a routine examination for possible bariatric surgery. It was reported a thyroid imaging reporting and data system 5 left lobe nodule, measuring 4 × 5 × 8 mm, without any suspicious lymph nodes. Figure 1*A* shows the initial aspect of the thyroid nodule–small, hypoechoic nodule, irregular borders, and microcalcification. The patient was advised on the suspicious nature of the nodule, although its size would preclude the need for fine needle aspiration biopsy.^1^ The patient wanted to acknowledge the nodule’s origin and asked for the fine needle aspiration biopsy that came out Bethesda VI. Three options were offered: surgery, radiofrequency ablation (RFA), and active surveillance. The latter was discarded since the patient wanted to “do something about the tumor.” She refused lobectomy since hypothyroidism was a risk. The patient opted for RFA of the nodule. The procedure occurred under local anesthesia in an outpatient fashion. A 5-mm active tip needle and the moving-and-shot technique were used, in order to achieved nodule’s complete ablation. Figure 1*B* shows the early aspect of the ablated nodule–an increase of the nodular area, loss of borders, highly hypoechoic.

### Follow Up of Radiofrequency Ablation of a Thyroid Nodule

**Fig. 2:**
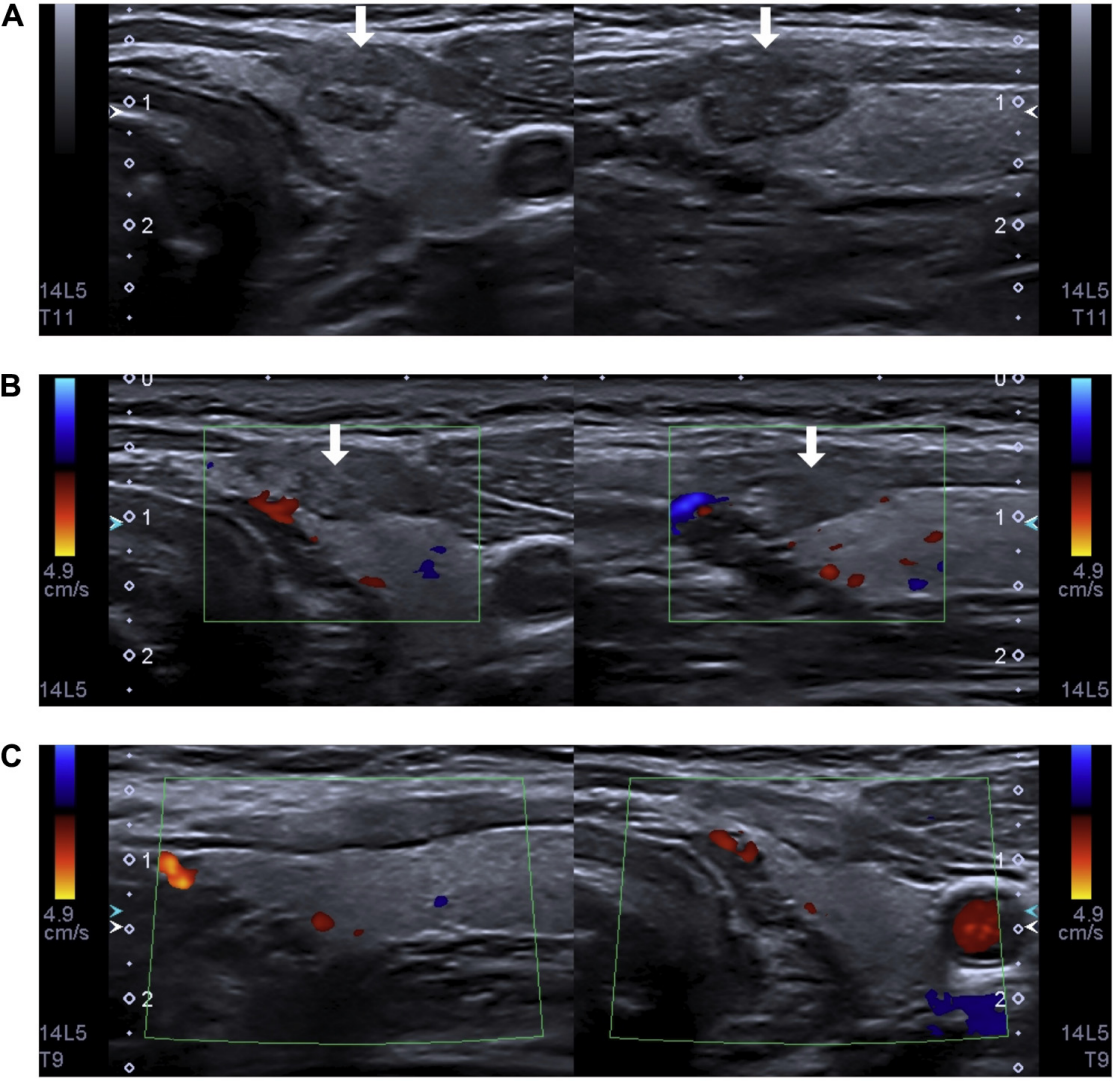

One-month follow-up, the ultrasound exam demonstrated an enlarged irregular, hypoechoic lesion. Figure 2*A* shows the first month post-radiofrequency ablation. After 3 months of follow-up, we could not identify mild strap muscle fibrosis with thyroid parenchymal lesion over the treated area. An ablation margin was necessary since the nodule was adjacent to the capsule. Figure 2*B* shows the third month after ablation–thyroid parenchyma without identifiable lesion, edema, and fibrosis over strap muscle. Finally, the inflammatory process decreased and no lesion could be discerned. Figure 2*C* shows the ninth month after ablation–complete disappearance of the nodule.

RFA of malignant thyroid nodules is a relatively recent option for patients unfit for surgery or refusing it. The most typical indication is nodules <1 cm in the largest size, albeit thyroid nodules up to 2 cm are amenable.^2^ Additionally, the position of the nodule is paramount since the ablation of such lesions needs a safety margin that would assure the complete destruction of all malignant cells. Thus, even small nodules adjacent to critical areas must not be approached by RFA. Nodules close by the recurrent laryngeal nerve posterior face of the thyroid are unsuitable for RFA. Nodules near but dissectible through hydrodissection, the trachea, the strap muscles, or the carotid can be carefully selected for RFA.^3^

Concurrently, the follow-up of the ablated nodules is unique. The ablated area will experience an increase in size and exacerbation of the suspicious ultrasound features in the early follow-up. The size reduction will initiate within the first couple of months and occur until the first year, with a total disappearance rate over 90% and a reduction rate of 99.3%.^4^

In conclusion, the use of RFA for malignant thyroid nodules is possible, but size and position considerations are advisable and particular aspects of the follow-up need to be acknowledged.
